# Prediction of Non-Muscle Invasive Papillary Urothelial Carcinoma Relapse from Hematoxylin–Eosin Images Using Deep Multiple Instance Learning in Patients Treated with Bacille Calmette–Guérin Immunotherapy

**DOI:** 10.3390/biomedicines12020360

**Published:** 2024-02-03

**Authors:** Julius Drachneris, Mindaugas Morkunas, Mantas Fabijonavicius, Albertas Cekauskas, Feliksas Jankevicius, Arvydas Laurinavicius

**Affiliations:** 1 Department of Pathology and Forensic Medicine, Institute of Biomedical Sciences, Faculty of Medicine, Vilnius University, 03101 Vilnius, Lithuania; arvydas.laurinavicius@vpc.lt; 2 National Center of Pathology, Affiliate of Vilnius University Hospital Santaros Klinikos, 08406 Vilnius, Lithuania; 3 Clinic of Gastroenterology, Nephrourology and Surgery, Institute of Clinical Medicine, Faculty of Medicine, Vilnius University, 08406 Vilnius, Lithuania; mindaugas.morkunas@santa.lt (M.M.); albertas.cekauskas@santa.lt (A.C.); feliksas.jankevicius@santa.lt (F.J.); 4 Center of Urology, Vilnius University Hospital Santaros Klinikos, 08406 Vilnius, Lithuania; m.fabijonavicius@santa.lt

**Keywords:** digital image analysis, bladder cancer, deep learning, cancer prognosis, survival prediction, feature extraction

## Abstract

The limited reproducibility of the grading of non-muscle invasive papillary urothelial carcinoma (NMIPUC) necessitates the search for more robust image-based predictive factors. In a cohort of 157 NMIPUC patients treated with Bacille Calmette–Guérin (BCG) immunotherapy, we explored the multiple instance learning (MIL)-based classification approach for the prediction of 2-year and 5-year relapse-free survival and the multiple instance survival learning (MISL) framework for survival regression. We used features extracted from image patches sampled from whole slide images of hematoxylin–eosin-stained transurethral resection (TUR) NPMIPUC specimens and tested several patch sampling and feature extraction network variations to optimize the model performance. We selected the model showing the best patient survival stratification for further testing in the context of clinical and pathological variables. MISL with the multiresolution patch sampling technique achieved the best patient risk stratification (concordance index = 0.574, *p* = 0.010), followed by a 2-year MIL classification. The best-selected model revealed an independent prognostic value in the context of other clinical and pathologic variables (tumor stage, grade, and presence of tumor on the repeated TUR) with statistically significant patient risk stratification. Our findings suggest that MISL-based predictions can improve NMIPUC patient risk stratification, while validation studies are needed to test the generalizability of our models.

## 1. Introduction

Non-muscle invasive papillary urothelial carcinoma (NMIPUC) is the most common type of urinary bladder cancer, with variable clinical courses ranging from very indolent tumors with low risks of relapse after transurethral resection (TUR) to highly aggressive tumors with very high risks of early relapse and progression to muscle-invasive bladder cancer [1]. Based on clinical and pathological data, NMIPUC patients are stratified into risk groups, where patients with a higher risk of relapse are treated with Bacille Calmette–Guérin (BCG) immunotherapy to reduce the risk of cancer relapse. However, even after immunotherapy, over 30% of patients suffer disease relapse and, in some cases, progression [2]; meanwhile, a delayed cystectomy leads to worse cancer-specific survival [3]. Better risk stratification is needed to select patients for more aggressive treatment strategies, which might prevent very high-risk patients from developing metastatic disease and thus reduce cancer-related mortality.

Tumor grading is among the most critical factors for NMIPUC patient risk stratification [4,5,6]. However, the limited reproducibility of NMIPUC grading [7] requires a search for more robust methods of tumor histology assessment. Advances in digital whole slide image (WSI) and deep-learning (DL) techniques open new possibilities to extract computational biomarkers based on tumor histology [7].

Recently, several studies showed promising results employing deep-learning-based automatic tumor grading [8,9,10]. Jansen et al. developed a fully automated tumor detection and grading network [8]. Subsequently, Wetteland et al. developed an automatic diagnostic tool predicting tumor grade with an average F1 score of 0.91 for high- and low-grade tumors [9]. Zhang et al. developed a deep-learning-based system that not only outperforms pathologists but also produces descriptions of histological findings in the NMIPUC tumor tissue [10]. However, this approach depends on histological features defined by pathologists. Therefore, it only reproduces current medical knowledge. To evade this limitation, Lucas et al. performed a study predicting 1-year and 5-year relapses of NMIPUC using features extracted by a pre-trained VGG16 neural network, reaching 0.61 and 0.67 accuracy, respectively, thus demonstrating that the prediction of NMIPUC patient outcomes directly from hematoxylin and eosin (H&E) is possible.

Intratumoral heterogeneity poses a problem in the assessment of tumors in the WSI of full-face histology sections. Previous studies have shown the importance of identifying focal areas of higher grade for the assessment of NMIPUC risk with tumors having both high- and low-grade areas showing clinical behavior intermediate between the high-grade and low-grade tumors [11,12,13,14]. The attention-guided multiple instance learning (MIL) framework addressed this problem by adding an attention layer, helping us to focus on the most important image areas while also addressing the variability in the number of image patches [15]. Furthermore, the deep attention-guided multiple instance survival learning approach in lung and colorectal tumors developed by Yao et al. uses complete survival data (survival time and censoring data), thus better representing patients’ outcomes in comparison to the assessment of relapse-free survival (RFS) in a specific timeframe (e.g., one year) [16].

Here, we present a study on the prediction of NMIPUC relapse using an attention-guided deep MIL framework in a cohort of 157 patients treated with BCG immunotherapy. This rather uniformly treated patient cohort limits the spectrum of the tumors to intermediate and high risk, thus focusing the research question on the clinical setting where more aggressive patient treatment is in consideration.

## 2. Materials and Methods

### 2.1. Patient Cohorts

We retrospectively collected clinical and pathological data of all 230 bladder cancer patients who received BCG immunotherapy at VUH SK between 2009 and 2020. For predictive modeling, 166 patients were selected according to the following inclusion criteria: diagnosed with papillary urothelial carcinoma at pTa or pT1 stage; completion of full (6-week) BCG induction therapy; availability of tumor resection material collected prior to the BCG induction (within one year before induction); and complete clinical, pathological, and follow-up data (time to tumor relapse or last follow-up if the patient did not experience relapse) available. Survival data were censored at 5 years of follow-up to exclude cases with more likely development of new primary tumor rather than true relapse of primary tumor. Data also included findings of repeated TUR (reTUR), which was performed in 121 patients.

**Figure 1:**
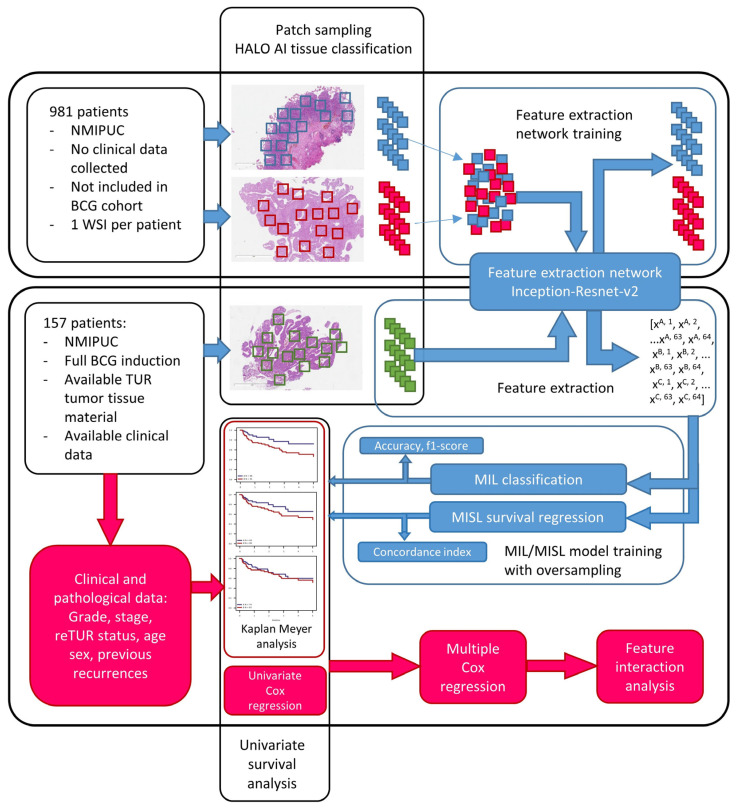
Study design chart. Upper panel: feature extraction network training on 981 patients H&E WSIs. Lower panel: prognostic modeling on image data (blue arrows) and clinical, pathological data together with best-selected image-based model (red arrows).

To train the feature extraction network, we recruited an independent training cohort of 981 NMIPUC patients from the same period who were not part of the study cohort. Pathology diagnosis of NMIPUC was the only selection criterion; no clinical or additional pathology data were collected. This strategy for training on a sizable, independent cohort was selected to develop a feature extraction network that yields more generalizable features (Figure 1).

H&E-stained tumor tissue slides from patients in both cohorts were reviewed by the pathologist (JD). The most representative single tissue slide per patient was selected for further analyses. All slides were digitized at 20× magnification (0.5 µm per pixel) using an Aperio^®^ AT2 DX scanner (Leica Aperio Technologies, Vista, CA, USA).

### 2.2. Tissue Area Classification and Artifact Exclusion

**Figure 2:**
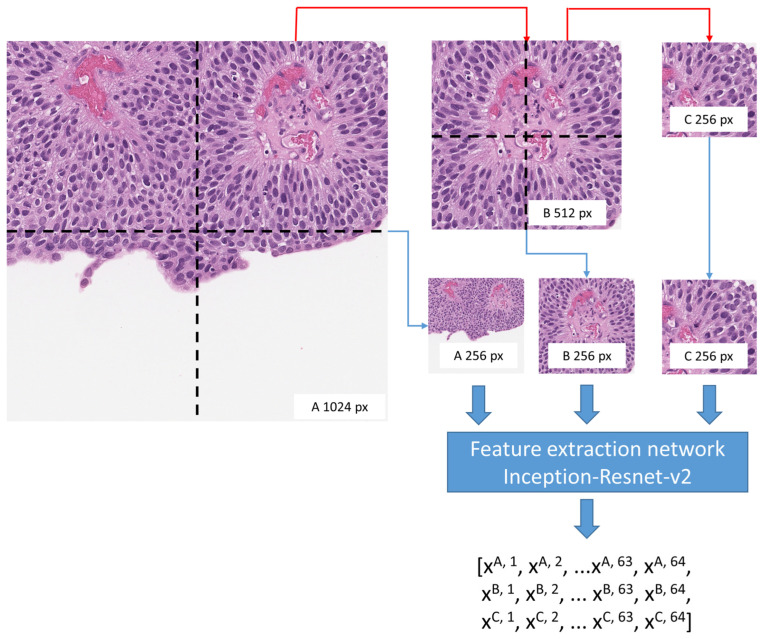
Hierarchical patch subdivision for multiresolution analysis. A 1024-pixel patch subdivided into four 512-pixel patches which were further subdivided into 256-pixel patches. Also, 1024- and 512-pixel patches were resized to 256-pixel using the memory efficient resize method (bilinear interpolation resampling) in the OpenCV Python framework. Patches were passed to feature extraction network, and for each 256-pixel patch, the corresponding larger-sized patch feature vectors were concatenated.

To classify tumor tissue into ‘stroma’, ‘epithelium’, and ‘artifacts’ compartments (Figure 2), we trained the HALO^®^ AI (Indica Labs, Albuquerque, NM, USA) Densenet v2 classifier using manual annotations provided by the pathologist (JD) in BCG-treated patient cohort. The annotations were created using a built-in HALO^®^ AI annotation tool via the user-friendly graphical user interface. The image data within the annotated regions are automatically incorporated into the model training pipeline through HALO^®^ AI’s native methods, eliminating the need for manual data management. The ‘artifacts’ class was incorporated to exclude areas of coagulation, necrosis, hemorrhage, or calcifications that could potentially interfere with further analyses. The quality of tissue classification masks produced by HALO^®^ AI was visually assessed by a pathologist (JD). Following initial tissue classification and artifact exclusion, due to the very low area (less than two mm^2^) of the remaining tumor, 9 cases were excluded, leaving 157 for further analyses. The clinical and pathological data of these patients are summarized in Table 1.

### 2.3. Image Data Sets

Our study employed 1024-, 512-, and 256-pixel-sized patches and a multiresolution patch approach. In computer vision, it is commonplace to employ image patches with dimensions that are integer powers of 2, as these sizes align seamlessly with the hierarchical subdivision framework, enabling a comprehensive analysis of the image’s hierarchical structure. The decision to commence with a patch size of 256 pixels was deemed appropriate due to its ability to achieve a harmonious balance between the extraction of fine-grained details and the preservation of the image’s overall context. This selected patch size was also shown to be optimal for medical image analysis in the study by Rukundo [17]. Figure 2 visually demonstrates the efficacy of patches of varying sizes in capturing distinct tissue characteristics. Smaller patches (256 pixels) are expected to put emphasis on tumor cytology details, whereas larger patches (1024 pixels) represent tissue microarchitecture. To prepare image data for multiresolution analysis, a hierarchical subdivision technique was employed, facilitating the seamless integration of patches at disparate resolutions. To prepare image data for multiresolution analysis, we employed a hierarchical subdivision. Initially, 1024-pixel-sized image patches were extracted from the WSIs. To ensure the analysis focused on relevant tissue regions, we selected patches with at least 50% tissue content as determined by the ratio of total pixels belonging to the ‘stroma’ or ‘epithelium’ classes and the total number of pixels in a corresponding patch in the predicted HALO^®^ AI classifier mask (as described in Section 2.2). This tissue content control mechanism is a crucial safeguard against artifacts and non-tissue regions, enhancing the reliability and relevance of our findings. Subsequently, the 1024-pixel patches meeting the tissue content criterion were subdivided into 512-pixel patches, followed by a further division into 256-pixel patches. This procedure resulted in a hierarchical series of image patches at different resolutions (see Figure 2), providing multiscale representations for subsequent analyses.

Patches in each resolution were assigned into 3 clusters according to stroma and epithelium content defined by HALO^®^ AI tissue classifier (C1 cluster being predominantly (>50%) composed of stroma, C2 cluster—having <50% of stroma and <50% of epithelium, and C3 being predominantly (>50%) composed of epithelium). Similarly, multiresolution patches were assigned into clusters according to 1024-pixel patch epithelium–stroma content.

We associated demographic information (sex, age), clinical data (treatment modalities, history of tumor recurrence, status of repeated transurethral resection (TUR), location, number and size of tumors, and relapse-free survival data), and pathological details (tumor grade, stage, association with carcinoma in situ) with each WSI in our study cohort.

### 2.4. Image Feature Extraction

**Figure 3:**
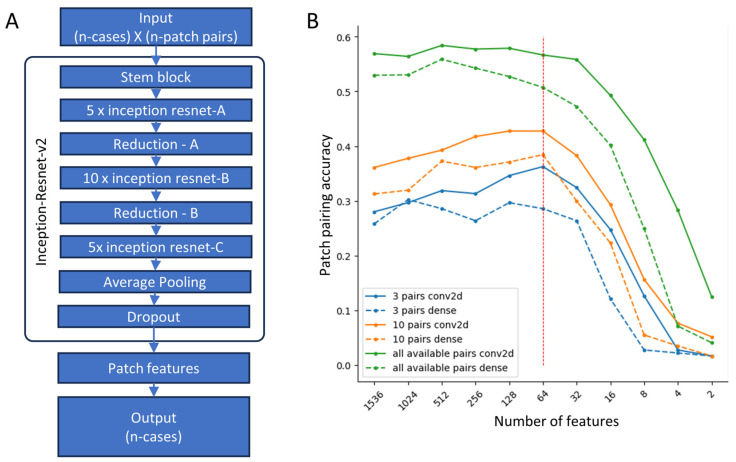
(**A**) Feature extraction network architecture based on Inception-Resnet-v2 with variation in patch counts in the input layer and variations in patch feature layer (variations in architecture dense layer vs. convolutional 2D, and number of features from 1536 to 2). (**B**) Feature extraction network optimization. The best results were achieved using all available patch pairs in comparison with three and ten pairs, and convolutional 2D architecture outperformed dense layer architecture constantly. Decreasing number of features to 64 did not hinder the performance of network, while further decrease led to fast deterioration in performance.

To predict patient outcomes from readily available data, routinely H&E-stained histology tissue WSIs, we converted the sampled image patches into feature vectors. This feature extraction was performed according to the method published by Rawat et al. [18]. Similarly, we designed our feature extraction model based on InceptionResNetV2 architecture and tasked it to assign the same identity index correctly to all patches sampled from the same patient WSI. To train the feature extraction network, we composed a dataset of neighboring patch pairs sampled from training cohort WSIs. One patch from each pair was reserved to train the feature extraction network, while the second was only used to validate the training (resulting in a 0.5 training validation data ratio).To optimize the feature extraction network, we have run experiments with 256 pixel-sized patches employing variations in the dataset and the mode of feature extraction (Figure 3). We tested the patch pair matching accuracy for the different numbers of patch pairs per WSI used to train feature extraction models. We prepared datasets by extracting 3 patch pairs, 10 patch pairs, and 100 patch pairs per single WSI. When 100 patch pairs per WSI were unavailable, we employed all available patch pairs. To reduce the length of extracted feature vectors, we employed an additional compression layer—the last layer before the decision layer, with two variations utilizing the 2D convolutional or the dense layers and conditioning these layers with a different number of output features—1536, 1024, 512, 256, 128, 64, 32, 16, 8, 4, and 2.

### 2.5. Deep Multiple Instance Learning (MIL) to Predict Patient Relapse

**Figure 4:**
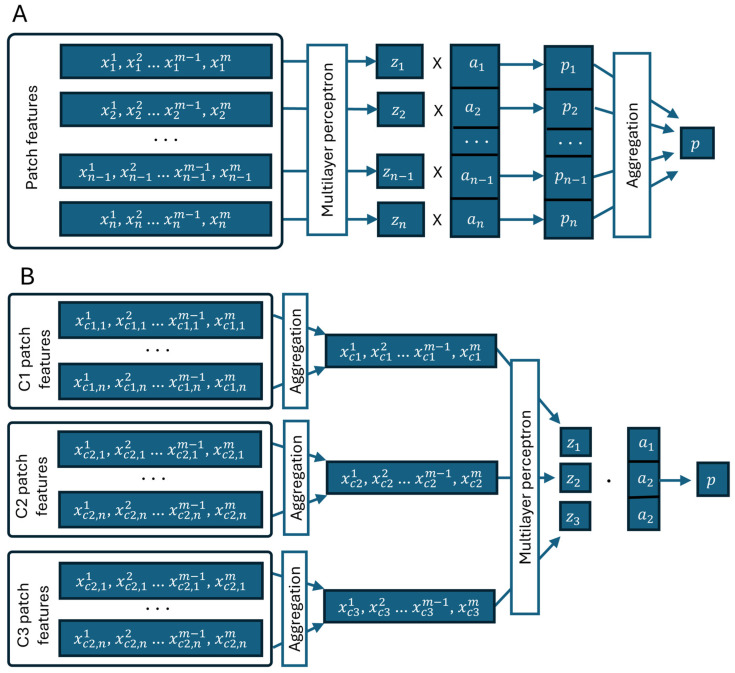
Architecture of MIL and MISL frameworks. (**A**) MIL framework takes feature vectors (number of features—*m*) of all available (number of patches—*n*) patches followed by multilayer perceptron(MLP) neural network. All individual predictions of MLP (*z*) for patches are element-wise multiplied by attention layer values (a), and all individual patch outputs (*p_1_–p_n_*) are aggregated to single case level prediction p. (**B**) MISL framework is first aggregating feature vectors in every individual cluster to single vector of length m. Then, dot product of cluster level predictions and attention layer produces single case level prediction *p*.

We adopted the MIL implementation as proposed by Ilse [15] to train a simple convolutional neural network (CNN) model (Figure 4). This classical MIL assumption involves feeding the network with batches of data extracted from WSIs and provides the ability of binary classification based on clinical data categories. We modified the original method to accept image feature vectors instead of image patches. Therefore, each MIL batch comprised a set of feature vectors from image patches originating from the same WSI, and the batch label was derived from the associated clinical data value for the corresponding WSI. The model is capable of handling bags of varying lengths. Training involves a small CNN with the Adam optimization algorithm, terminating when validation loss remains unchanged for 200 epochs. As per the original method, an attention-based MIL pooling layer is incorporated before the model’s final layer. The objective function is the negative log-likelihood of the Bernoulli distribution.

To evaluate the performance of our MIL model, we implemented a 5-fold cross-validation scheme, randomly dividing the patient cohort into five equal folds. The model was trained on four folds and evaluated on the remaining fold, repeating this process for each fold. This procedure provided a robust assessment of the model’s generalizability to unseen data.

To balance the MIL training dataset due to a low number of patients with relapse (positive class), we employed minority class oversampling. Oversampling was achieved by repeatedly drawing random positive cases from the minority pool to balance the class distribution.

### 2.6. Deep Multiple Instance Survival Learning (MISL) to Predict the Risk of Relapse

An image feature vector-based standard MIL assumption for binary classification can be adapted to model patient survival by utilizing a loss function based on survival probability. For MISL, we have adopted a method published by Yao et al. [16]. The MISL method adapts the negative partial log-likelihood as a loss function and an average concordance index as a training metric.

To train the MISL model effectively for patient survival prediction, we designed an oversampling technique that specifically addresses the imbalance in the patient survival status distribution. By augmenting the minority class, which represents patients with poorer survival outcomes, our oversampling technique ensures that the model is adequately exposed to the diverse patterns associated with shorter survival times.

The technique involves identifying the longest follow-up time (tmax = 1826 days) among all patients in the cohort. Subsequently, we artificially extend the follow-up period for each patient to match tmax, ensuring consistent evaluation of survival status across patients. Next, we define a fixed time interval (tstep = 30 days) to check the survival status of each patient at regular intervals. A cohort-wide survival matrix (T) with dimensions m x n is constructed, where m represents the number of patients, and n represents the number of tsteps. The matrix is filled row-wise by assigning the appropriate survival status (left-censored, event, or right-censored) for each patient at each tstep. The resulting oversampled MISL training set, represented by the completed survival matrix T, provides a balanced representation of patient survival status and facilitates effective training of the MISL model. We trained the MISL model in a 3-fold cross-validation setting.

### 2.7. Survival Analysis

Both MIL and MISL predictions from each fold of a k-fold cross-validation were aggregated to reconstruct the entire cohort’s survival statistics and were assessed by Kaplan–Meyer survival analysis. A one-sided log-rank test was used to assess the difference between patient groups in MIL and MISL experiments. To compare different outcome prediction methods, we have stratified patients into two groups according to cut-off value with lowest MISL-predicted log-rank test *p*-value. We performed multiple Cox regressions to analyze MISL model prediction performance in the context of other clinical features. We have used partial Akaike information criterion (AIC) for assessment of the model’s prediction error and concordance index (C-index) to assess predictive performance of the models. We used Kaplan–Meyer survival analysis in patient groups defined by other prognostic features. Additionally, differences in the distribution of prediction values between patient subgroups were assessed using Kruskal–Wallis and Man–Whitney U tests where applicable.

## 3. Results

### 3.1. Optimization of Feature Extraction Network

The goal of optimization was to reduce the dimensionality of image feature space by removing redundancy. We aimed to retain a low number of highly informative features. The accuracy of patch pairing was the only metric used to measure the performance of feature extraction models. Overall, the highest accuracies were obtained by models trained on a maximum number of patch pairs per WSI and models utilizing convolutional feature compression (see Figure 3). Models using the dense feature compression layer in all scenarios resulted in a lower patch pairing accuracy. The accuracy of models trained on 100 patch pairs per WSI remained stable as the convolutional feature extraction layer was compressed from its original width of 1536 features down to 64 features. However, further compression to 32 features and below resulted in a rapid decline in accuracy. In this setting, the dense feature extraction resulted in an even earlier decline in patch pairing accuracy. Even though the accuracy of models trained on lower numbers of patch pairs per WSI was significantly lower in the whole range of extracted features, the effect of compression of the feature extraction layer was quite the opposite—in this setting, the patch pairing accuracy increased in the range from 1536 features down to 64 features retained.

We ran the optimization experiments on a dataset prepared from 256-pixel-sized image patches. Based on these observations, for our further experiments, we utilized image feature vectors produced by models trained on 100 patch pairs per WSI using convolutional feature compression and a 64-feature-long image feature vector.

### 3.2. Prediction of Patient Relapse by Deep MIL

Table 2 summarizes the results of different image resolution deep MIL models’ cross-validation metrics of patient relapse prediction and the log-rank statistics of survival differences between the two predicted groups.

The best results of 2-year relapse prediction were obtained with image features extracted from down-sampled 1024-pixel patches (resized to 256 pixels). Although this experimental setting allowed for the highest F1 score (0.654) and accuracy (0.672), the survival differences between the predicted groups were not statistically significant (log-rank *p*-value 0.257). The features extracted from the multiscale and the 256-pixel-sized patches also allowed reasonable prediction results, achieving both F1 scores and accuracies above 0.6. However, the relatively high log-rank values of 0.208 and 0.441, respectively, suggest lower predictive reliability.

The 5-year relapse prediction analysis did not reveal any significant results, with an accuracy above 0.5 achieved only using the features extracted from down-sampled 512-pixel patches (resized to 256 pixels). However, none of the experiments reached at least a 0.5 F1 score.

In general, models using features derived from 256-pixel-sized patches and larger patches resized to 256 pixels showed a tendency to perform better in both 2-year and 5-year relapse prediction. However, none of these experiments yielded a statistically significant stratification of the patients in the survival analysis. Thus, these findings should be received with caution.

### 3.3. Prediction of Risk of Relapse by Deep MISL

The MISL results are summarized in Table 3. Only two models using a multiresolution approach and features extracted from 1024-pixel-sized patches were able to stratify patients into risk groups with similar statistical significance in survival difference (log-rank *p*-value < 0.05), with the multiresolution approach showing a slightly higher C-index (0.574 vs. 0.564) and slightly better performance on the validation splits, hence better generalizability of the models’ performance. In general, features obtained from smaller patches (256-pixel size or larger patches resized to 256-pixel size) performed worse in survival prediction. In contrast, the MISL models trained with features extracted from downsized 512-pixel patches (resized to 256-pixel) reached the highest (0.579) C-index; however, these models did not stratify patients into statistically significant risk groups.

### 3.4. Clinicopathological Variables and Cox Regression Analysis

**Figure 5:**
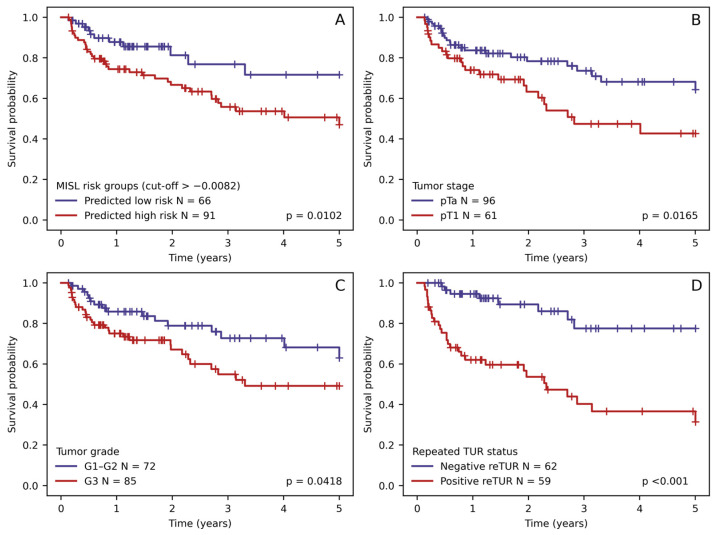
Kaplan–Meyer RFS plots stratified according to (**A**) stratified multiple instance survival learning (MISL) risk prediction results, (**B**) tumor stage (pTa vs. pT1), (**C**) tumor grades (1973 grading system), and (**D**) presence of tumor in reTUR.

Significant relapse hazard differences were observed only by a repeated TUR tumor grade and stage (reTUR) with hazard ratios 5.018, 1.9902, and 1.8545, respectively (Table 4 and Figure 5). These features were selected for multiple Cox regression together with stratified MISL prediction.

Notably, tumor grade demonstrated a significant difference in relapse hazard stratification (*p*-value 0.0451) only when assigned using the WHO 1973 classification system but not when assessed using the WHO 2004 system (*p*-value 0.1807).

**Figure 6:**
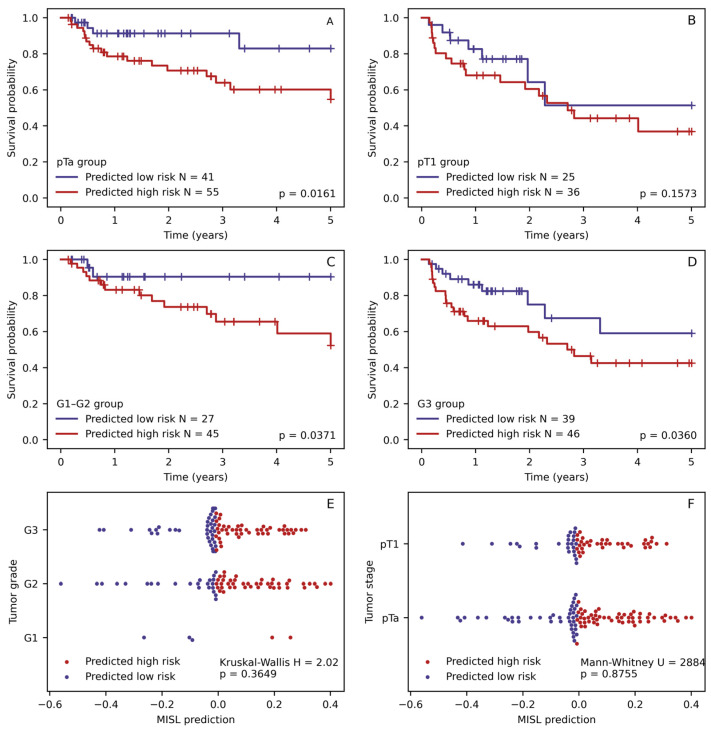
Kaplan–Meyer RFS plots of patient survival stratified by multiple instance survival learning (MISL) in patient stage (**A**,**B**) and grade (**C**,**D**) subgroups. MISL prediction value distribution between tumor grade (**E**) and stage (**F**) groups.

We generated all possible combinations of selected features and evaluated them using multiple Cox regression. Three Cox regression models yielded significant individual features (*p*-values < 0.05). All three models (see Table 5) consisted of two independent predictive features combining MISL-based risk stratification with one of the selected clinicopathologic features (stage, grade, and reTUR status). This finding prompted a further investigation of the relationship between MISL prediction and histology-derived features, as well as an evaluation of MISL-based risk stratification in the tumor grade and stage subgroups. We found that MISL prediction was capable of successfully substratifying the patients in the pTa, pT1 stage, and G1–G2 grade groups. Additionally, we found a similar distribution of MISL prediction scalar values between the tumor stage and grade groups (*p* = 0.876 and *p* = 0.365, respectively), supporting the independence of this feature (see Figure 6).

## 4. Discussion

In our study, we have developed a DL-based NMIPUC risk stratification model, which can improve the prediction of tumor relapse in the setting of BCG immunotherapy. By comparing two DL approaches for patient risk prediction, we found that models based on time-dependent survival probability data performed better than models based on a dichotomous prediction of the relapse event during a given time period. In other words, survival data, including the status of relapse and the exact time of the event, provide a more precise definition of patient outcome, thus improving predictions of the more aggressive behavior of the tumor.

The difference in the accuracy of prognostic performance between the 1973 WHO and the 2004/2016 WHO grading system may be explained by the fact that the threshold between G2 and G3 grades defined by the 1973 WHO system might be more relevant in our cohort of patients than groups defined by 2004/2016 WHO. Since our study includes predominantly patients in intermediate or higher risk groups, the latter system separates only a small subgroup of low-grade NMIPUC tumors. This observation supports the current NMIPUC grading approach by reporting both grading systems in the diagnostic workup. However, since our study includes predominantly patients with intermediate or higher risk groups, the latter system separates only a small subgroup of low-grade NMIPUC tumors. The limited prognostic performance of 2004/2016 WHO might be attributed to class imbalance in our patient cohort.

From other clinical and pathological variables, only the tumor stage and findings of residual tumor at reTUR showed prognostic significance in univariate and multivariable models. We have reported these findings in our previous study, which focused on tumor-infiltrating CD8 lymphocytes in a very similar patient cohort [19]. While the tumor stage is widely used for the assessment of patient risk, the adverse effects of positive reTUR were so far not utilized as a prognostic indicator despite strong evidence to support this [20,21,22,23].

By comparing MIL models that predict tumor relapse in 2- and 5-year periods, we found that models based on the prediction of relapse in a shorter period performed better despite having fewer events. This observation contradicts the results reported by [24], where they used deep learning to predict relapse in 1-year and 5-year periods. Our finding may be attributed to the fact that more aggressive tumors tend to relapse earlier, and these tumors might have a higher degree of architectural and cytological atypia at the histological level, which makes these tumors more straightforward to identify with image analysis.

A comparison of the models based on different patch sizes revealed that larger patch size experiments and multiresolution approaches tended to perform better. The performance advantage of the multiresolution method might be explained by a more comprehensive representation of the properties of tumor tissue extracting both cytological details and architectural features at lower and higher resolutions, respectively. On the other hand, the difference in the performance of single-resolution inputs might indicate that architectural features (large patches) might be more representative of tumor biological behavior.

The values of the MISL multiresolution approach did not show a significant difference in the distribution between the stage and grade groups of patients; therefore, they are not associated with these known indicators and are likely to represent a novel computational biomarker to predict relapse. Furthermore, the stratification of the patients by the MISL indicator in most subgroups (see Figure 6) retained significant differences in the relapse probabilities. Overall, this highlights the potential to improve risk stratification based on histology image analysis data.

The most important limitation of our study is the lack of external validation. Also, our cohort included patients from a single center, which might cause relative homogeneity in patients’ management, H&E staining quality, and evaluation by the pathologist, thus limiting the generalizability of our findings. We have tested several variations in image patch sizes and a number of patches used for feature extraction network training. However, there are many possible variations in DL models and their hyperparameters. Yet, it was out of the scope of this study, so we implemented the models as they were published in the previous studies. We look forward to collaborating with other laboratories in the field while planning the validation of these findings in our prospective patient cohort and refining the implementation of MISL to facilitate more explainable predictions. 

## 5. Conclusions

We found that DL-based pathology image analysis can extract additional prognostic information on NMIPUC patient outcomes, independent of current clinical and pathologic criteria. Our MISL model enabled an improved prediction of disease relapse within the grade and stage subsets of the patients on BCG therapy. Additionally, we found that models based on full survival data were superior to dichotomous classification tasks, thus guiding further work in predictive modeling to more effective methodologies. Further studies are needed to assess the generalizability and explainability of our models. 

## Figures and Tables

**Table 1:** Summary of clinical and pathological data.

Characteristic	Value (%)
Patients	157 (100%)
Age, years	
Median (range)	69.8 (33–89)
Gender	
Male	129 (82.2%)
Female	28 (17.8%)
RFS time, months	
Median (range)	16.6 (1–60)
Recurrences	47 (29.9%)
Tumor grade WHO 2004	
Low	12 (7.6%)
High	145 (92.4%)
Tumor grade WHO 1973	
G1	5 (3.1%)
G2	67 (42.7%)
G3	85 (54.1%)
pT stage	
Ta	95 (61.1%)
T1	61 (38.9%)
Carcinoma in situ association	8 (5.1%)
Positive reTUR	59 (48.8%)
Recurrent tumor	48 (30.6%)
Multiple tumors	79 (50.3%)
Tumor size > 30 mm	43 (31.9%)
EORTC risk group	
Intermediate	77 (50.3%)
High	71 (46.4%)
Very High	5 (3.3%)

**Table 2:** Performance of multiple instance learning models predicting relapse in 2-year and 5-year periods by different input image (patch) sizes.

Patch Size (Pixels)	2-Year Relapse Prediction			5-Year Relapse Prediction		
	F1 Score	Accuracy	Log-Rank *p*-Value	F1 Score	Accuracy	Log-Rank *p*-Value
Multiresolution	0.618	0.622	0.208	0.422	0.446	0.916
1024	0.590	0.610	0.423	0.412	0.438	0.427
1024 resized to 256	0.654	0.672	0.257	0.476	0.492	0.486
512	0.572	0.566	0.134	0.472	0.492	0.579
512 resized to 256	0.592	0.592	0.323	0.494	0.502	0.613
256	0.626	0.620	0.441	0.480	0.490	0.268

**Table 3:** Performance of multiple instance survival learning models by different input image (patch) sizes compared by concordance index and log-rank test *p*-values.

Patch Size (Pixels)	Concordance Index	Log-Rank *p*-Value
Multiresolution	0.574	0.010
1024	0.564	0.007
1024 resized to 256	0.562	0.687
512	0.569	0.053
512 resized to 256	0.579	0.126
256	0.532	0.095

**Table 4:** Univariate Cox regression results of clinicopathological data and stratified multiple instance survival learning prediction results.

Feature	Hazzard Ratio	*p*-Value
Positive reTUR	5.018	0.0001
pT1 stage	1.9902	0.0187
MISL prediction > −0.0082	2.1849	0.0237
G3 (1973 grading system)	1.8545	0.0451
High grade (2004 grading system)	2.6389	0.1807
Association with carcinoma in situ	1.8076	0.2586
EORTC high or very high risk group	1.3548	0.3012
Multiple tumors	1.3395	0.3489
Recurrent tumor	1.255	0.4549
Muscle presence in TUR	1.2098	0.6668
Sex	1.1822	0.683
Tumor size > 30 mm	1.1281	0.7376

**Table 5:** Multiple Cox regression models with *p* values of individual features < 0.05.

Features	Hazard Ratio	95% CI	*p*-Value
Model: positive reTUR + MISL prediction. AIC = 302.40; C-index = 0.73			
Positive reTUR	4.907	2.245–10.726	<0.001
MISL prediction	2.181	1.058–4.499	0.035
Model: G3 grade (WHO 1973) + MISL prediction. AIC = 418.59; C-index = 0.64			
G3 grade (WHO 1973)	2.026	1.105–3.716	0.023
MISL prediction	2.374	1.202–4.688	0.013
Model: pT1 stage + MISL prediction. AIC = 418.76; C-index = 0.63			
pT1 stage	1.969	1.109–3.495	0.021
MISL prediction	2.164	1.099–4.263	0.026

## Data Availability

Data presented in this study can be obtained from the author upon request. These data are not available to the public due to permit restrictions.
